# Transcriptome Sequencing and Annotation for the Jamaican Fruit Bat (*Artibeus jamaicensis*)

**DOI:** 10.1371/journal.pone.0048472

**Published:** 2012-11-15

**Authors:** Timothy I. Shaw, Anuj Srivastava, Wen-Chi Chou, Liang Liu, Ann Hawkinson, Travis C. Glenn, Rick Adams, Tony Schountz

**Affiliations:** 1 Institute of Bioinformatics, University of Georgia, Athens, Georgia, United States of America; 2 The Jackson Laboratory, Bar Harbor, Maine, United States of America; 3 Department of Statistics, University of Georgia, Athens, Georgia, United States of America; 4 School of Biological Sciences, University of Northern Colorado, Greeley, Colorado, United States of America; 5 Department of Environmental Health Science, University of Georgia, Athens, Georgia, United States of America; CSIRO, Australia

## Abstract

The Jamaican fruit bat (*Artibeus jamaicensis*) is one of the most common bats in the tropical Americas. It is thought to be a potential reservoir host of Tacaribe virus, an arenavirus closely related to the South American hemorrhagic fever viruses. We performed transcriptome sequencing and annotation from lung, kidney and spleen tissues using 454 and Illumina platforms to develop this species as an animal model. More than 100,000 contigs were assembled, with 25,000 genes that were functionally annotated. Of the remaining unannotated contigs, 80% were found within bat genomes or transcriptomes. Annotated genes are involved in a broad range of activities ranging from cellular metabolism to genome regulation through ncRNAs. Reciprocal BLAST best hits yielded 8,785 sequences that are orthologous to mouse, rat, cattle, horse and human. Species tree analysis of sequences from 2,378 loci was used to achieve 95% bootstrap support for the placement of bat as sister to the clade containing horse, dog, and cattle. Through substitution rate estimation between bat and human, 32 genes were identified with evidence for positive selection. We also identified 466 immune-related genes, which may be useful for studying Tacaribe virus infection of this species. The Jamaican fruit bat transcriptome dataset is a resource that should provide additional candidate markers for studying bat evolution and ecology, and tools for analysis of the host response and pathology of disease.

## Introduction

Bats are an ancient and diverse group [1] and are the second largest taxonomic group of mammals with more than 1,200 identified species among the 5,499 known mammals [2], [3]. Bats are the only mammals to have evolved powered flight, which has allowed dispersal across all continents other than Antarctica. Bats are critical components of ecosystems, serving as major predators of insects, pollinating flowers and dispersing seeds of keystone plant species worldwide. The body sizes of bats range from less than 2 gm with 8 cm wingspans to more than 1 kg with 2 m wingspans. Most contemporary species of bats are insect-, nectar-, or fruit-eaters, but about 1% are carnivores, including fish-eating and blood-drinking species.

The evolutionary origin of bats remains controversial [4], [5]. In early work, bats were thought to be closely related to rodents and primates [6]. Bats are now established within Laurasiatheria; however, the placement of bats within Laurasiatheria has been difficult to resolve because the major groups diverged from one another within a relatively short period of time [7]. Different placements recently hypothesized for bats include: (A) sister to Perissodactyla (horse) [8]; (B) sister to Cetartiodacyla (cattle+dolphin) [5], (C) sister to Perissodactyla+Cetartiodacyla (horse, cattle, dolphin) [9], (D) sister to Ferungulata (cattle+dolphin, dog+horse) [4], [10] and (E) the Pegasoferae hypothesis which places bat with Perissodactyla and Carnivora (horse+dog) [11] (see [5] for a review).

Two bat genomes have been sequenced to date [12], the little brown bat (*Myotis lucifugus*, 7× coverage*)* and the large flying fox (*Pteropus vampyrus*, 2.6× coverage), but neither has been extensively annotated. These species represent the two major clades within bats: the microbats and megabats. Transcriptome sequencing for another megabat species, the Australian flying fox (*Pteropus alecto*), has recently been published [13]. Thus, a transcriptome for a microbat species is needed.

Many highly pathogenic viruses are hosted, or suspected to be hosted, by bat reservoirs, including ebolaviruses, Marburg virus, Hendra virus, Nipah virus, rabies virus and coronaviruses [2]. In total, more than 100 viruses have been isolated from, or detected in, bats of dozens of species, yet many of the viruses that cause disease in humans cause little or no disease in the bats. Significantly, the great majority of bat species have not been examined for infectious agents and are, thus, likely underappreciated as reservoir hosts. The continued encroachment of humans upon bat habitat and bat migrations caused by climate change may lead to novel infectious diseases among humans and livestock. Moreover, some infectious diseases cause significant morbidity and mortality in bats that could have dramatic impacts on population numbers and cascading ecological effects [14]. Thus, the study of bats and their infectious agents is an important but neglected aspect of zoonotic and wildlife disease research.

Jamaican fruit bats (*Artibeus jamaicensis*) are one of the most common bats in the tropical Americas, ranging from the Caribbean Islands, tropical South and Central America, Mexico and the Florida Keys [15]. The Jamaican fruit bat is a microbat in the family Phyllostomidae, which contains 56 genera and 192 species. They are a frugivorous generalist and fig specialist of medium size; about 80 mm in length with a wingspan of 130 mm and mass of about 50 grams. They can readily fly 20 km per night, although they typically maintain a smaller home range as long as food is available, and can live 9 years or more in the wild. Females typically produce two offspring per year and provide maternal care for about 50 days, with pups reaching adult body weight by about 80 days. Several microbes of interest have been isolated from or detected in Jamaican fruit bats, including *Histoplasma capsulatum*, *Trypanosoma cruzi*, eastern equine encephalitis virus, Mucambo virus, Jurona virus, Catu virus, Itaporanga virus and Tacaiuma virus, suggesting the species may be an important reservoir and vector of infectious diseases [15]–[18]. It is unknown what diseases these pathogens may cause in bats.

Tacaribe virus (TCRV) was isolated from 11 artibeus bats (6 *A. lituratus*, 5 *A. jamaicensis*) in the late 1950s in and near Port of Spain, Trinidad [19]. TCRV was the first arenavirus isolated in the Americas and during the next decades other arenaviruses with substantial similarity to TCRV were identified that cause the South American hemorrhagic fevers (SAHF) [20], [21]. The known reservoir hosts for all other arenaviruses are rodents, making TCRV exceptional for its repeated isolation from artibeus bats. Because exhaustive searches for evidence of other potential reservoir hosts of TCRV failed to suggest another reservoir species [19], [22], it has been suspected that artibeus bats are reservoirs of the virus. However, recent work by us demonstrated that TRLV-11573, the only remaining isolate of TCRV, causes a fatal infection resembling the SAHF in Jamaican fruit bats, one of the species from which TCRV was isolated, or is cleared without disease, suggesting this species is not a suitable reservoir host for TCRV and the virus may be a significant pathogen for bats [23].

Because of the equivocal role of artibeus bats as a reservoir host species for TCRV, and because of the similarities with human SAHF, the Jamaican fruit bat may be a novel model for studying the pathology of the disease. However, as an unusual, non-model organism, very little is known about its physiology, immunology or host response to TCRV. No antibodies are available with known specificity to Jamaican fruit bat proteins, which dramatically limits its usefulness. To address some of these deficiencies, we have performed transcriptome sequencing and analysis of spleen, lung, kidney and poly-IC-stimulated primary kidney cells to identify genes of interest for assessing the host response to TCRV infection.

## Results

### Sequence Assembly and SNP detection

More than 240,000 454 reads and 142 million Illumina reads were obtained (Table 1). The reads were submitted to Short Read Archive (SRA) under SRR539297 and SRR538731. Reads from lung, kidney, and poly-IC-stimulated primary kidney cell libraries were pooled for a combined *de novo* assembly using the 454 gs Assembler program, yielding 6,450 contigs. For the Illumina spleen sequences, we first corrected reads using the SOAPdenovo correction tool and further assembled them using SOAPdenovo, yielding 214,707 contigs. A total of 367,317 SNPs and 44,679 indels were detected through GigaBayes. At least 16 reads covering a site were required to ensure the SNP was of high quality. Using TGICL, a combined assembly of the 454 and Illumina contigs was constructed that contained 102,237 contigs with N10, N50, and N90 of 3,882 bp, 1,004 bp, and 289 bp, respectively.

**Table 1:** Assembly statistics.

	454 Lung and Kidney	Illumina SpleenPaired End	Combined Assembly
**Raw reads #**	241327	142351486	–
**Corrected Reads**	–	119262966	–
**Contigs/Scaffold**	6450	108065	102237
**Total number of BP in Contigs/Scaffold**	6668393	82606450	71067811
**N50**	2899	641	1004

### Localization of Contigs

**Figure 1:**
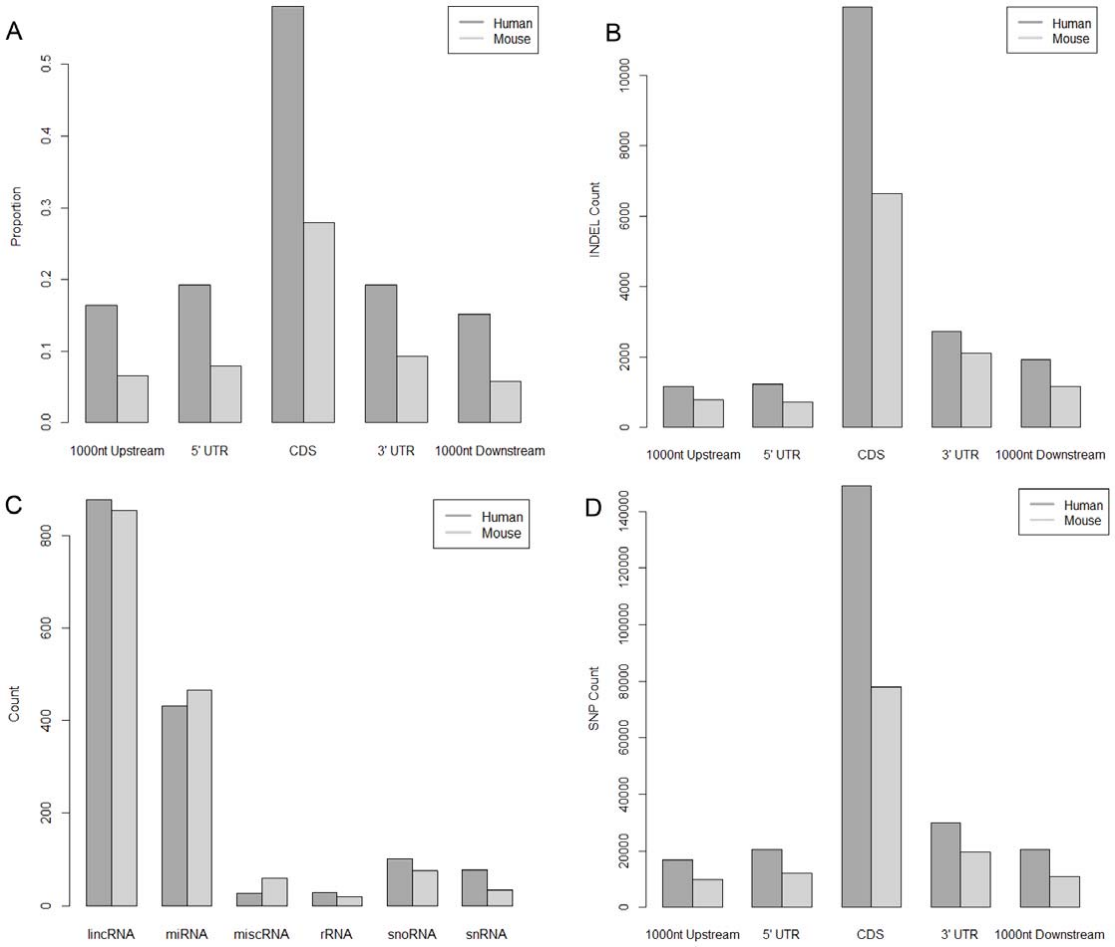
Distribution of mapped contigs. Histograms displaying the proportion of contigs mapped to particular features of protein coding genes of human and mouse (UTR is the untranslated region, and CDS is the coding sequence). The upper panel (A) displays the raw count and the lower panel (B) normalized values (the proportion discovered relative to how many could be discovered within each category). The raw count of SNPs (C) and Indels (D) mapped to particular features of protein coding genes of human and mouse.

Human and mouse genomes were used as references to estimate the distribution of bat contigs within known gene transcripts. Human and mouse genomes were chosen for completeness of their annotations. Genomic features were divided into 5 groups: 1 Kb upstream of 5′ UTR, 5′ UTR, CDS (coding sequence), 3′ UTR, 1 Kb downstream of 3′ UTR. We found 58.03% and 23.18% mapped CDS region for human and mouse genome respectively (Figure 1A). Because we performed transcriptome sequencing, we expected a majority of the sequences to map to CDS and UTR regions of the genome. Many RNA genes were also mapped, including long noncoding RNAs and a substantial number of microRNAs (Figure 1B). Annotation was concentrated on identifying microRNAs because they could be cross validated through their RNA secondary structure features. To further obtain a confident set of microRNA sequences, a microRNA prediction pipeline was used to cross validate the BLAST mapping of prediction. In the process, 42 confident microRNA candidates were found that have been deposited within MirBase [24], [25]. We present the list of predicted microRNAs as Table S1. Mapping the predicted SNPs on the genomic features indicates that the vast majority of SNPs are in the CDS region (Figure 1C–1D). Although humans and mice are both outside Laurasiatheria the relatively fast rate of molecular evolution of mice is expected to result in more differences between bats and mice than bats and humans [26]–[29]. The presence of sequences mapped 1 Kb upstream or downstream of the known transcript indicated possible alternative splicing from human and mouse transcripts.

### GO Localization of all Contigs

**Figure 2:**
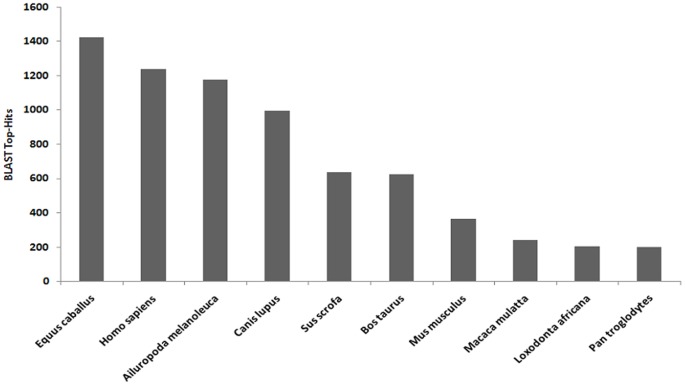
Species with more than 100 top hits from B2G.

**Figure 3:**
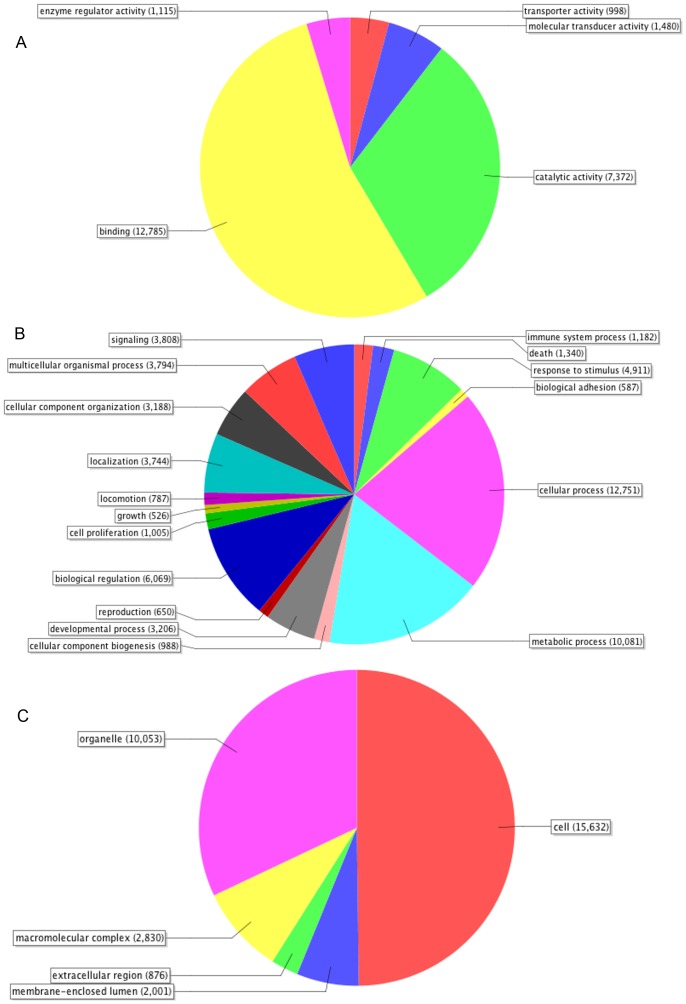
B2G annotation for Molecular Function, Biological Process, and Cellular Component Level 2.

**Figure 4:**
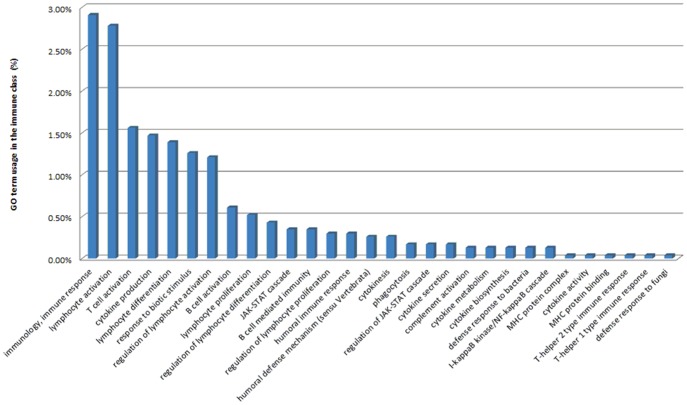
Distribution of immune genes at the GO slim level based on CateGOrizer.

BLAST2GO was used to functionally annotate contigs. A total of 20,020 contigs (19.58% overall) had significant matches to known proteins in the NCBI non-redundant protein (nr) database. Horse and human were identified as the top two species with best BLAST hits for bat contigs (Figure 2). The BLASTX annotation process is biased by the completeness of the annotation for each respective genome; therefore, despite the lack of a completely annotated horse genome, a high similarity between bat and horse genomes was apparent. The human genome is well annotated, which explains the high number of BLAST hits between bat and human. The GO annotation divides the functional annotation into three main components: biological process, cellular process, and molecular [30]. A majority of the annotated genes encoding proteins that function within a cell or organelle are involved in metabolic and cellular processes. The primary molecular functions of these genes are catalytic and binding activities (Figures 3A–C). A total of 466 immune-related genes were annotated by BLAST2GO. These immune genes include toll-like receptors, cytokines, transcription factors, kinases and several chemokine receptors. In addition, CateGOrizer was used to categorize the immune class using the GOslim database, resulting in 30 categories representing a broad range of immune activities (Figure 4). The immune response and lymphocyte activation genes represented the largest proportion of theses transcripts.

### Unannotated Contigs

**Figure 5:**
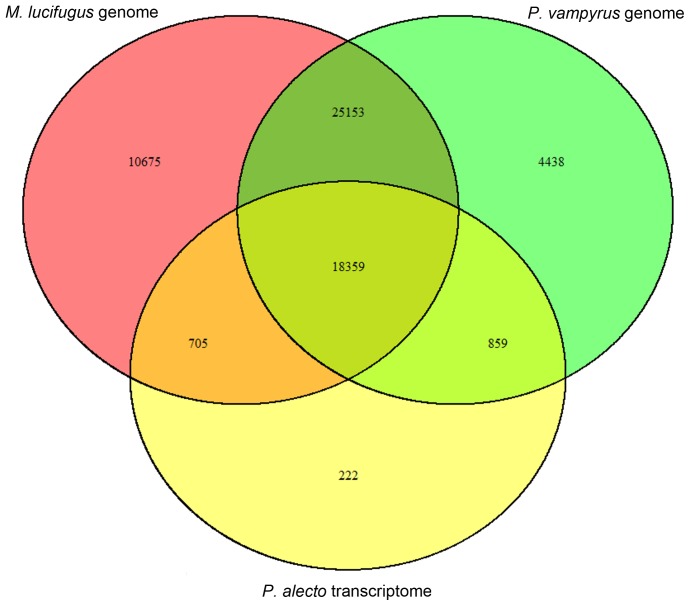
Venn Diagram comparison of unannotated transcript mapped to *Myotis lucifugus* genome, *Pteropus vampyrus* genome, and *Pteropus alecto* transcriptome.

There were 82,218 unannotated contigs. A total of 16,869 sequences had open reading frames longer than 300 nt; 5,417 were identified through BLASTP to the nr database with E-value<1e^−3^. For the remaining contigs, 54,892 mapped to the assembled *Myotis lucifugus* genome, and 48,809 mapped to the assembled *Pteropus vampyrus* genome. There were 20,145 contigs that mapped to *Pteropus alecto*, Australian flying fruit bat, and 18,359 that overlapped between genomic and transcriptome sequences for all three datasets (Figure 5). Through this process, we were able to account for 65,828 (80%) unannotated contigs.

### Mapping Bat Contigs in Immunological Pathways

**Figure 6.KEGG:**
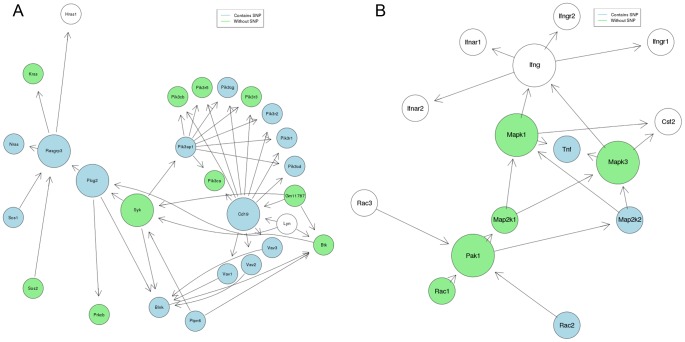
KEGG Mapped Genes. A graphical representation for two KEGG pathways: (A) B cell receptor signaling pathway and (B) natural killer cell-mediated cytotoxicity. Because the original KEGG graph is much larger, we only present the center genes and genes neighboring these central nodes. If mapped, genes representing nodes were either highlighted with light green or light blue colors. Genes highlighted with light blue contain SNPs.

The completeness of genes mapped to immunological pathways was examined using human and mouse as reference species. Based on the ortholog data obtained, all contigs were mapped onto immune system related KEGG pathways (Table 2) and determined that many genes were missing from these pathways. This could be due to the low expression within bat tissues or due to the overly stringent e-value cutoff of 1e^−20^ during reciprocal BLAST annotation that we chose to limit the number of false positives. A KEGGgraph visual representation of contigs mapped onto the mouse pathway was generated (Figures S1, S2, S3, S4, S5, S6, S7, S8, S9, S10, S11, S12, S13, S14, S15, S16, S17, S18). Pathways involved in the adaptive immune response, T and B cell signalling pathways, generally had more mapped genes than did those involved in innate response or natural killer (NK) cell-mediated cytotoxicity pathways (Figures 6A and 6B). The NK cell cytotoxicity pathway appears to have almost half of its genes missing, whereas the B cell receptor pathway appears to have most of its genes present.

**Table 2:** Comprehensively mapped genes on the KEGG pathway.

Pathways	Contigs Mapped toHuman Pathway	Proportion of MappedHuman Pathway	Contigs Mapped to Mouse Pathway	Proportion of Mapped Mouse Pathway
**Toll-like receptor signaling pathway**	73	0.715686275	66	0.653465347
**RIG-I-like receptor signaling pathway**	49	0.690140845	43	0.623188406
**Cytokine-cytokine receptor interaction**	133	0.501886792	93	0.379591837
**Cell adhesion molecules_CAMs**	87	0.654135338	73	0.489932886
**Complement and coagulation cascades**	36	0.52173913	28	0.368421053
**Intestinal immune network for IgA production Apoptosis**	78	0.735849057	72	0.620689655
**Fc gamma R-mediated phagocytosis**	80	0.85106383	78	0.866666667
**Chemokine signaling pathway**	131	0.693121693	121	0.654054054
**Leukocyte transendothelial migration**	84	0.724137931	79	0.658333333
**Jak-STAT signaling pathway**	93	0.6	76	0.496732026
**mTOR signaling pathway**	51	0.980769231	50	0.943396226
**MAPK signaling pathway**	211	0.787313433	199	0.742537313
**T cell receptor signaling pathway**	94	0.87037037	88	0.8
**ErbB signaling pathway**	73	0.83908046	68	0.781609195
**B cell receptor signaling pathway**	68	0.906666667	62	0.815789474
**Natural killer cell mediated cytotoxicity**	66	0.485294118	58	0.464
**VEGF signaling pathway**	64	0.842105263	55	0.723684211
**TGF-beta signaling pathway**	59	0.702380952	59	0.694117647

### Substitution Rate Estimation

Nucleotide substitution in the coding region can be synonymous or non-synonymous. The ratio between the rate of synonymous (dS) and non-synonymous mutation (dN) can be used to infer the degree of selection operating on the system. We used the human genome as a reference for dN/dS calculations because the human genome is well annotated. Reciprocal BLAST was used to identify human, mouse, and bat orthologs. MACSE was used to generate codon alignments. The alignments were trimmed for excessive gap codon triplets, and PAML was used to calculate dN/dS for each gene. When genes are highly conserved, synonymous mutations (dS) tend to be estimated as 0, resulting in a larger dN/dS ratio, therefore those results were removed from the analysis. After filtering, dN/dS results were obtained for 14,717 genes. The majority of the genes have close to zero dN/dS with clear evidence of purifying selection, a feature common among mammalian genes [31]–[33]. For investigation of positive selection, Tang et al. [34] have argued that a dN/dS threshold of greater than 1 for positive selection might be overly stringent. Because of this, a dN/dS cutoff of 0.7 was chosen to investigate genes that might be experiencing weak purifying selection. A total of 138 genes above the 0.7 threshold were found (Table S2).

**Figure 7:**
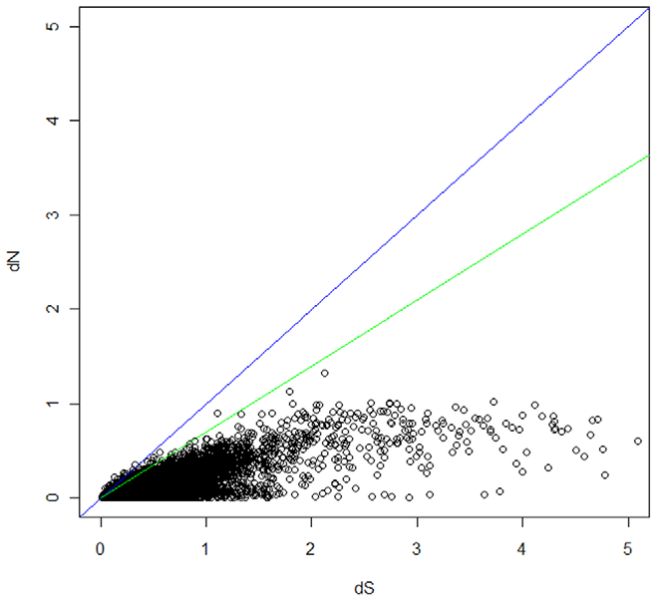
Substitution estimation scatter plot. We calculated the nonsynonymous mutation rate (dN) and synonymous mutation rate (dS) using orthologous genes between bat and human. Two lines were drawn representing the two dN/dS cutoffs of 0.7 (green) and 1.0 (blue).

For genes with evidence of positive selection, 32 exceeded the 1.0 dN/dS threshold (Figure 7). Through annotation by DAVID [35], [36], there were 14 genes involved in transcriptional activation and regulation processes. There were 9 genes associated with cellular signaling. In particular, we found DNA-damage-inducible transcript 4 (DDIT4) gene with dN/dS 1.4053; this protein is involved in the mTOR signaling pathway and it regulates cell growth and promotes neuronal cell death [37], [38]. Ectodysplasin A (EDA), involved with cytokine:receptor interaction pathways, had a dN/dS value of 1.23.

### Resolving Species Tree for Bat within Laurasiatheria

**Figure 8:**
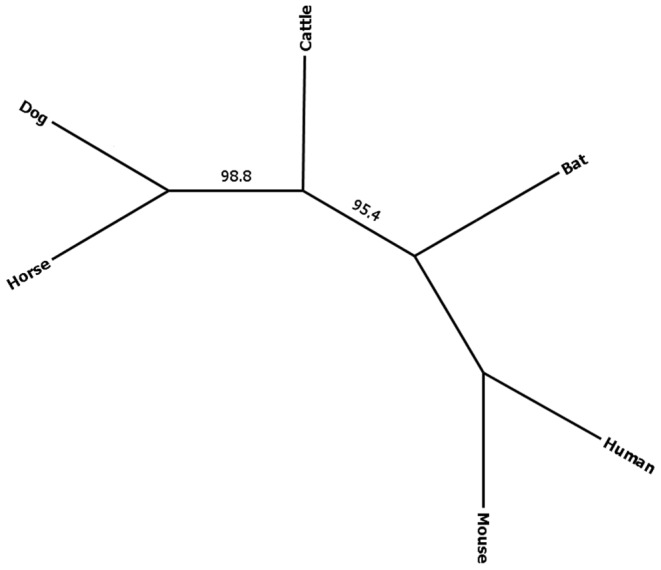
Unrooted species tree from the orthologous dataset across six *Boreoeutheria* mammals. The species tree was generated from 2378 gene loci. There was 95% bootstrap support for placing bats (Chiroptera) sister to Perissodactyla, Cetartiodactyla, and Carnivora.

The phylogenetic placement of bats within Laurasiatheria is still unresolved. Through reciprocal BLAST, we identified 8,785 putative orthologs across mouse, rat, cattle, horse and human (Table S3). Afterward we filtered out alignments with greater than 5% gap, the 2,378 genes remaining were used to construct 500 iteration multilocus bootstrap species tree (see methodology). This resulted in a highly supported species tree placing bat sister to the clade containing cattle, horse, and dog (Figure 8).

## Discussion

The Jamaican fruit bat transcriptome described here is a major new resource for genetic studies of bats. This bat is an important seed dispersing and pollinating species found in most of the tropical Americas. It is likely susceptible to infectious diseases, could be a zoonotic reservoir and vector, and may be a suitable model for the pathogenesis of SAHF. Considering the importance of immunological functions in response to infections, we conducted a transcriptome assessment of genes from spleen, kidney and lungs so that genetic tools and methods can be used to study this species as well as other microbats.

Genes were identified that mapped to immune response pathways; based on CateGOrize classification of immune classes, we found 40 different immune classes. Recently, the transcriptome sequencing for the Australian black flying fox was performed [13]. Our data contain a greater proportion of lymphocyte related immune classes than does the flying fox’s transcriptome dataset. However, our dataset also contained a lesser proportion of cytokine related immune classes than the flying fox’s transcriptome dataset. Genes involved in adaptive immune response generally had more mapped genes compared to genes involved in innate responses. From Figures 6A and 6B, more genes were mapped to the B cell receptor signalling pathway than to the NK cell-mediated cytotoxicity pathway. This bias is likely due to the large number of B cells found in the spleen. Due to our stringent BLAST criteria, it is also possible that lowering the e-value threshold could obtain additional genes mapped but at the risk of more false positives. We deposited 42 microRNA genes for *A. jamaicensis* into MiRBase, and according to MiRBase this gene set is the first deposited bat microRNA genes.

Estimates of substitutions within the orthologous contigs found 32 genes with a dN/dS ratio>1. This ratio provides a guide for indicating potential genes that are under positive selection. Many genes were involved in transcriptional activation/regulation processes, suggesting potential differences in the transcriptional regulating architectures of bats and humans. The DDIT4 and EDA have a dN/dS ratio>1, suggesting these genes are under positive evolutionary selection. DDIT4 is involved in regulation of cell death and its positive selection suggests a potential difference in cell death regulation between human and bats; further analysis will need to be performed to verify the functional differences. Another potential positively selected gene, EDA is associated with ectodermal dysplasia type 1 [39], a disorder associated with abnormal development of physical structures, including skin, hair, nails, teeth, and sweat glands. We suspect the bat’s EDA gene could be used as a potential reference for future studies of the disorder.

For transcripts that failed to be identified by reciprocal BLAST searches, we predicted the ORF for the unannotated contigs and used BLASTP against the nr database to identify 5,349 unannotated contigs. For the remaining unannotated contigs, we used genomic data from *Myotis lucifugus* and *Pteropus vampyrus*, as well as transcriptome data from *Pteropus alecto* to identify additional unannotated contigs. Existing *Artibeus* contigs that were not present within the nr database, but overlapped among *Myotis* and *Pteropus* genomic and transcriptomic sequences indicated the possibility for bat specific transcripts. We also found contigs that mapped only to the *Myotis lucifugus* genome indicating the possibility for microbat specific contigs. In total, we were able to account for 80% of the unannotated trancripts, and the remaining unannotated transcripts likely include misassembled contigs, contigs not sequenced sufficiently in the other bats to be included in their genome assemblies, as well as a few transcripts specific to *Artibeus jamaicensis.* Many additional analyses are warranted to further refine the transcriptome information from *Artibeus* and other bats.

Phylogenomics is an important tool for resolving the Tree of Life, and this transcriptome data set provides an opportunity to study the evolutionary history of bats. Bats were once thought to be closely related to primates [6]; however, further work using molecular information placed them within Laurasiatheria [40]. Our finding of bat as sister to the clade containing horse, dog, and cattle is consistent with the recent study by McCormack et al. [4] and Zhou et al [10]. Here, we used 2,378 loci from a microbat and species tree analyses to obtain 95% bootstrap support, whereas McCormack et al. use 683 loci to obtain 64%bootstrap support. A recent study by Nery et al. [5] obtained a concatenated data from 3,733 loci from megabat with 100% bootstrap support and 1.0 posterior probability placing bat as sister to cattle. Our phylogenetic tree is less resolved than Nery et al., probably because we did not include the more limited transcriptome data available from dolphin and hedgehog. Maximum likelihood analyses are powerful, yet can lead to incorrect conclusions in certain situations, whereas species tree analyses are less powerful but more robust to well-known violations of the models used for maximum likelihood phylogenetic analysis, such as incomplete lineage sorting (see [5] and references therein). Additional work is clearly warranted, especially by using additional taxa, testing for convergence and specific violations of gene-tree models, and other sources of conflict among protein-coding genes and other portions of the genome.

A principal difficulty for identifying mechanisms of pathogenesis of the SAHF, in which the immune response may play a contributory role, is a lack of animal model resources that faithfully recapitulate human disease [41]. Although laboratory mice (*Mus musculus*) and rats (*Rattus norvegicus*), which have substantial experimental methodologies and reagents, can be infected with Junín virus (JUNV), the etiologic agent of Argentine hemorrhagic fever, the pathogenesis is markedly different than human disease. The guinea pig (*Cavia porcellus*) typically exhibits signs of disease that closely resembles human disease; however, there are few immunological or genetic tools for assessing the host response to infection. JUNV is also a BSL-4 and select agent, thus use of virulent strains is confined to only a few laboratories with highly specialized containment facilities. The pathogenesis of TCRV, a BSL-2 agent, in Jamaican fruit bats exhibits many similarities to the SAHF in humans, thus the use of transcriptome data could be useful for studying pathogenesis using a variety of new technologies, such as PCR arrays for pathway discovery, and for the development of antibodies to specific artibeus proteins that are important in the pathogenesis of disease.

The transcriptome resource provided will facilitate research into artibeus host responses to infectious agents, including mechanisms of pathogenesis of arenavirus disease and will also provide further resource for additional understanding for the bat species evolution and physiological development.

## Materials and Methods

### Ethics Statement

All procedures were approved by the UNC Institutional Animal Care and Use Committee (IACUC) and were in compliance with the USA Animal Welfare Act. UNC animal care and use committee approval number, 1207C-RA-B-15.

### Bats, Cells and RNA Extractions

Five bats from the University of Northern Colorado Jamaican fruit bat colony were used for this work. Male and female *A. jamaicensis* bats were euthanized by respiratory hyperanesthesia followed immediately by thoracotomy. Tissues were aseptically removed and flash frozen in liquid nitrogen for subsequent RNA extraction. Tissues were homogenized in Buffer RLT (RNEasy kit, Qiagen, Valencia, CA) containing 2ME using a Bead Beater and silicone beads. The homogenate was passed over a Qiashredder column prior to total RNA extraction according to manufacturer’s instructions.

For cell culture, one kidney from one bat was collected in serum-free HBSS and minced under aseptic conditions, then trypsinized (trypsin-versene) at room temperature in a sterile 50 ml trypsin flask. Cells were washed 3× in 10% FBS-EMEM, then seeded into a vented T-25 flask. The next day, unattached cells were removed and fresh 10% FBS-EMEM added. When cells approached confluence they were passaged with trypsin at a split ratio of 1∶4. Poly-IC was added to 50 µg/ml in two T-75 flasks containing 20 ml each of 10% FBS-EMEM and incubated for 6 hours, after which RNA was extracted according to manufacturer’s instructions (Qiagen).

### Sequencing

Total RNA was extracted using the RNeasy MinElute Cleanup Kit (Qiagen) and then shipped on dry ice to SeqWright (Houston, TX) for cDNA library construction and sequencing. RNA concentrations and quality were assessed by A260/A280 and A260/A230 absorbance values and agarose gel electrophoresis. A260/A280 values were all above 2.0 and A260/A230 were all above 1.9. Electrophoresis of the RNA samples demonstrated that 28S and 18S rRNA were not degraded. Libraries for the 454 were prepared from three tissues (kidney, lung, poly-IC-stimulated kidney cells). For 454 library construction, full-length cDNA was synthesized with two set of primers for driver and tester cDNA [42], [43]. Single-stranded cDNA was used for hybridization instead of double-stranded cDNA. Excess amounts of sense-stranded cDNA hybridized with antisense-stranded cDNA. After hybridization, duplex was removed by hydroxyapatite chromatography. Normalized tester cDNA was re-amplified with tester specific primer L4N. Driver cDNA was unable to amplify using L4N. An Illumina TruSeq RNA library was made from spleens according to manufacturer’s instructions. The libraries were then sequenced according to manufacturer’s recommendations: 454 using Titanium chemistry and Illumina using 2×100 nucleotide paired-end sequencing on a Hi-Seq 2000.

### Sequence Assembly and Polymorphism Detection

The 454 and Illumina libraries were assembled individually and also by combining both libraries. Bases from the 454 reads were called from the 454 generated sff file using Pyrobayes [44] and 454 gs assembler (version 2.5) was used to perform the assembly. SOAP denovo [45] (version 1.04) was used to assemble reads obtained from spleen (Illumina library). Only contigs greater than 200 bases were used in the final analysis. Prior to performing the combined assembly, duplicates from pre-assembled contigs of lung, kidney and spleen tissues were removed with CD-Hit [46] (cd-hit-2009-0427) at default criterion and then combined into longer fragments with TGICL [47]. GigaBayes [48] - a short-read SNP/indel discovery program was used to detect polymorphisms. SNP/Indel detection was performed for both libraries separately. To make SNP/indel predictions more reliable, we used the criterion that minor allele and major allele (alleles with fewer reads are minor alleles, and alleles with more reads are major alleles) occur at least twice and 8 times for 454 and Illumina libraries, respectively.

### Localization of Contigs

To identify the approximate relative position of conserved mammalian genes, we mapped the bat contigs on to the genome of Mouse mm9 and Human GRCh37 (downloaded from the UCSC genome browser) using BLAT v.34 [49] with a minimum score of 80 used as a filter. Coordinates of the protein coding genes were obtained from Ensemble (http://uswest.ensembl.org/index.html) Xenoref and gtf files. We also normalized the number of BLAT hits based on the total annotated transcript regions (1000 nt upstream of 5′ UTR, 5′ UTR, CDS, 3′UTR, and 1000 nt downstream of 3′UTR) that were present in the mouse and human.

### Precursor Micro RNA Predictions

To predict precursor-microRNA genes within assembled sequences, we downloaded precursor microRNAs for mouse, rat and human from miRBase [24], [25]. We performed a BLAST search focused on high quality candidates, hits with ≥95% sequence identity [50]. Based upon RNAfold [51] secondary structure prediction, we further filtered out sequences that did not possess any hairpin loop structure. Previously, it had been demonstrated that microRNAs tend to have deterministic folding [52] and, therefore, we used Unpaired Structural Entropy (USE) to evaluate the RNA secondary structure base pairing distribution (cutoff 0.83 USE score). MiR-abela, a support vector machine learning program [53], was used to cross validate the prediction. The final remaining unfiltered sequences are considered as highly confident microRNA candidates.

### Orthology Identification

Orthologous contigs (against human, mouse, dog, cattle and horse) were identified using the reciprocal BLAST (BLASTN) approach [54] as it has been found to be superior to sophisticated orthology detection algorithms [55]. A stringent cutoff of 1e^−20^ was used to separate paralogs from orthologs. cDNA sequences from human (Homo_sapiens.GRCh37.64.cdna.all.fa), mouse (Mus_musculus.NCBIM37. 64.cdna.all.fa), dog (Canis_familiaris.BROADD2.64.cdna.all.fa), cattle (Bos_taurus. UMD3.1.64.cdna.all.fa) and horse (Equus_caballus.EquCab2.64.cdna.all.fa) were obtained from the Biomart database (www.biomart.org).

### dN/dS Calculation

The substitution rate is inferred from orthologous genes between bat and mouse. Sequences were aligned using MACSE [56] and an in-house java script was used to trim/remove codon gap triplets from the alignment. Substitution rate was estimated using a maximum likelihood method implemented in the CODEML program of PAML 4.5 [57], [58]. The pairwise maximum likelihood analyses were performed in runmode-2. Estimated rates of non-synonymous to synonymous substitutions (dN/dS) were plotted as a scatter plot.

### Functional Annotation Through BLAST2GO and KEGG

Blast2GO [59] was used to functionally annotate contigs. A combined graph was generated for each GO category. To prevent overloading graphs, the sequence filter value was changed to 500 in all 3 categories (biological process, molecular function and cellular component). Functional annotation was performed separately for all assembled contigs present in the combined assembly. Based on CateGOrizer [60], we further classified the genes using the GO slim database immune classes.

The completeness of mapping the bat genes using Euarchontoglires as a reference was further examined through KEGG. To do this, we first downloaded the xml file of annotated KEGG pathways [61], [62]for human and mouse. To identify genes that are functionally important within KEGG pathways, KEGGgraph was used to represent a graph form of the KEGG pathway. We further used KEGGgraph to compute the relative betweenness centrality, which is the algorithmic representation of the involvement of a node within a network. We chose to set a cutoff of grabbing the top 4 nodes within each network, or selecting the top 4 functionally important genes within each pathway [63].

### ORF Identification

Open reading frame was predicted from the assembled contigs through the OrfPredictor web server (http://proteomics.ysu.edu/tools/OrfPredictor.html) [64]. A customized java program was used to parse through the prediction to identify sequences longer than 300 nt. To perform additional annotation of the predicted open reading frame we used BLASTP with an e-value of 1e^−3^ against the most recent nr database that is available from NCBI during our analysis (August 26^th^, 2012).

### Bat Genome Comparison

Using contigs that were functionally unannotated, we compared the Jaimacan fruit bat contigs against three other available bat sequence dataset. Myotis lucifugus and Pteropus vampyrus genomes were downloaded from the ncbi traceDB FTP server (ftp://ftp.ncbi.nih.gov/pub/TraceDB/). The Pteropus alecto transcriptome was obtained from Dr. A. Papenfuss [13]. An e-value threshold of 1e^−5^ was used to indicate BLAST hit. We then used an R package VennDiagram [65] for displaying the mapped unannotated contigs that overlapped between different bat genome and transcriptomes.

### Species Tree Analysis

To resolve the evolutionary relationship for the artibeus bat species, we filtered the putative bat orthologs between human, mouse, dog, cattle, and horse. Insectivores such as hedgehog and dolphin were not used in our analysis due to limited gene annotation in these taxa. To obtain the best multiple sequence alignment for each putative orthologs, we used AQUA’s pipeline for performing multiple sequence alignment; the pipeline consists of multiple sequence alignment through MUSCLE and MAFFT which is refined by RASCAL and assessed by NORMD [66]–[69]. A customized java program was used to filter alignments (obtained through AQUA) with greater than 5% gap per sequence. Additionally, we filtered for sequences that are at least>1,000 bp long. PHYML 3 was used to generate a maximum likelihood gene tree [70], [71]. MrAIC, a perl script wrapper for PHYML, was used to infer the best substitution model for each gene tree based on AIC, AICc, BIC, and Akaike weights [72]. AIC was used as the objective function since not much variation was observed across different objective function. NJst was used to calculate the unrooted species tree based on our gene trees [73]. A customized Rprogram is used for Performing a nonparametric bootstrap species tree through resampling nucleotides within loci as well as resampling the loci within the data set as described by Seo [74].

## Supporting Information

Figure S1 **B cell receptor signalling pathway.**
(TIF)

Figure S2 **Cell adhesion molecules.**
(TIF)

Figure S3 **Chemokine signalling pathway.**
(TIF)

Figure S4 **Complement and coagulation cascades.**
(TIF)

Figure S5 **Cytokine-cytokine receptor interaction.**
(TIF)

Figure S6 **ErbB signalling pathway**
(TIF)

Figure S7 **Fc gamma receptor-mediated phagocytosis.**
(TIF)

Figure S8 **Intestinal immune network for IgA production.**
(TIF)

Figure S9 **Jak-STAT signalling pathway.**
(TIF)

Figure S10 **Leukocyte transendothelial migration.**
(TIF)

Figure S11 **MAPK signalling pathway.**
(TIF)

Figure S12 **mTOR signalling pathway.**
(TIF)

Figure S13 **Natural killer cell-mediated cytotoxicity.**
(TIF)

Figure S14 **RIG-I-like receptor signalling pathway.**
(TIF)

Figure S15 **T cell receptor signalling pathway.**
(TIF)

Figure S16 **TGF beta signalling pathway.**
(TIF)

Figure S17 **Toll-like receptor signalling pathway.**
(TIF)

Figure S18 **VEGF signalling pathway.**
(TIF)

Table S1 **List of annotated microRNAs.**
(XLSX)

Table S2 **List of genes with greater than 0.7 dN/dS ratio.**
(XLSX)

Table S3 **List of orthologous genes across each species.**
(XLSX)
