# Supplementary figures and images for: Transcriptome Sequencing and Annotation for the Jamaican Fruit Bat (Artibeus jamaicensis)
Source: PLoS One. 2012 Nov 15;7(11):e48472. doi: 10.1371/journal.pone.0048472 (PMC3499531; doi:10.1371/journal.pone.0048472)

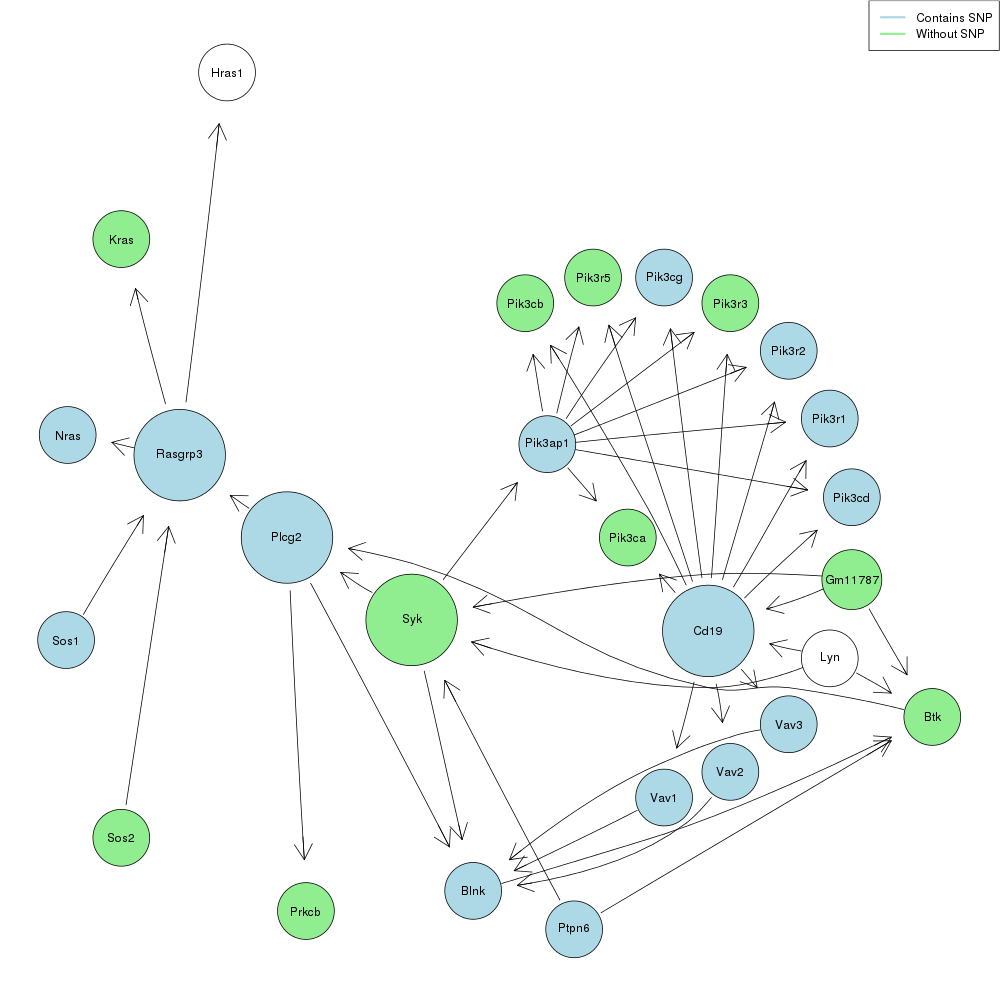

Supplement: Figure S1 — B cell receptor signalling pathway. (TIF) [file pone.0048472.s001.tif]

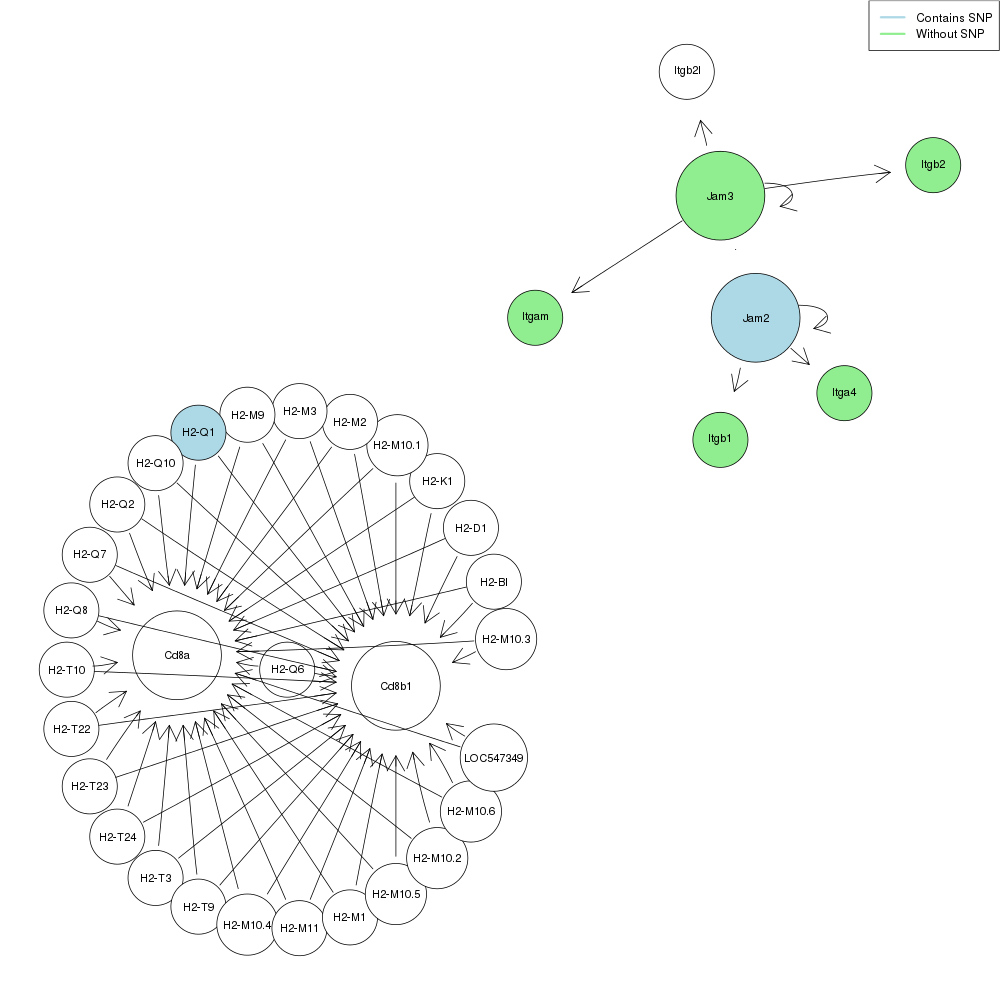

Supplement: Figure S2 — Cell adhesion molecules. (TIF) [file pone.0048472.s002.tif]

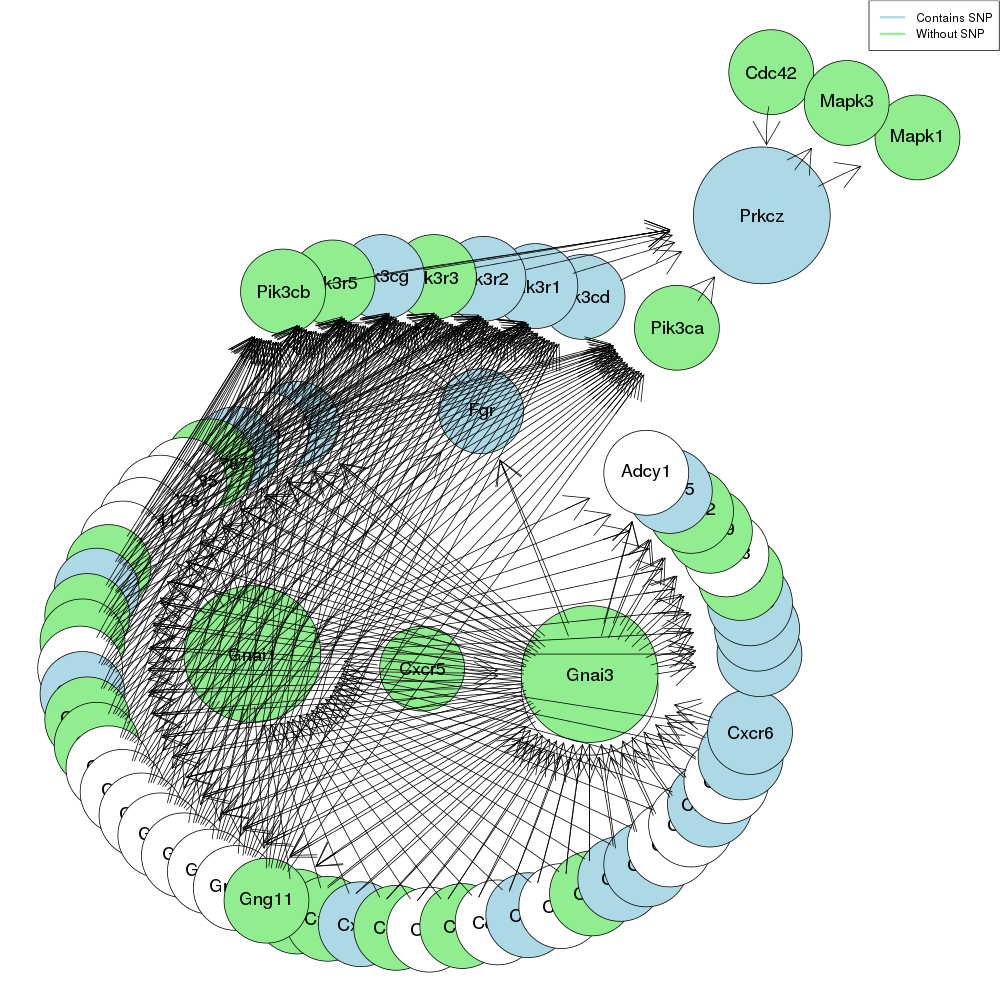

Supplement: Figure S3 — Chemokine signalling pathway. (TIF) [file pone.0048472.s003.tif]

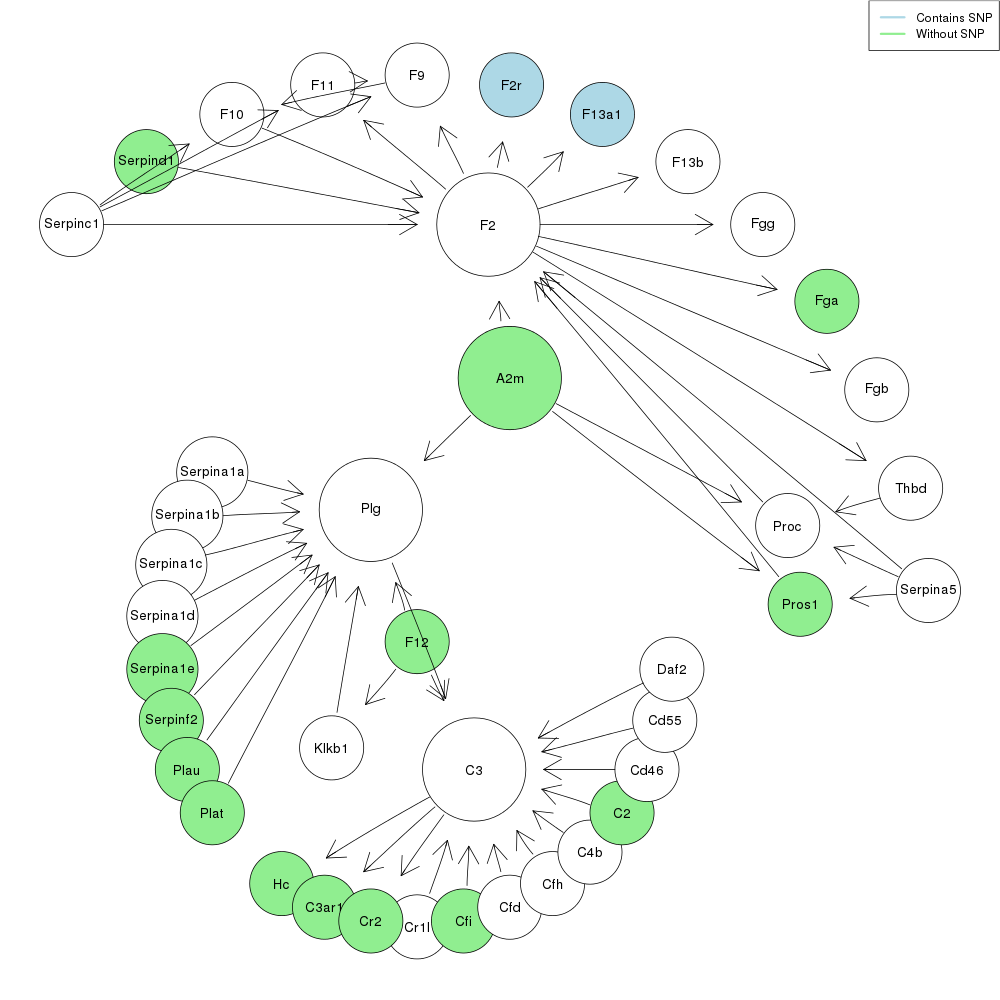

Supplement: Figure S4 — Complement and coagulation cascades. (TIF) [file pone.0048472.s004.tif]

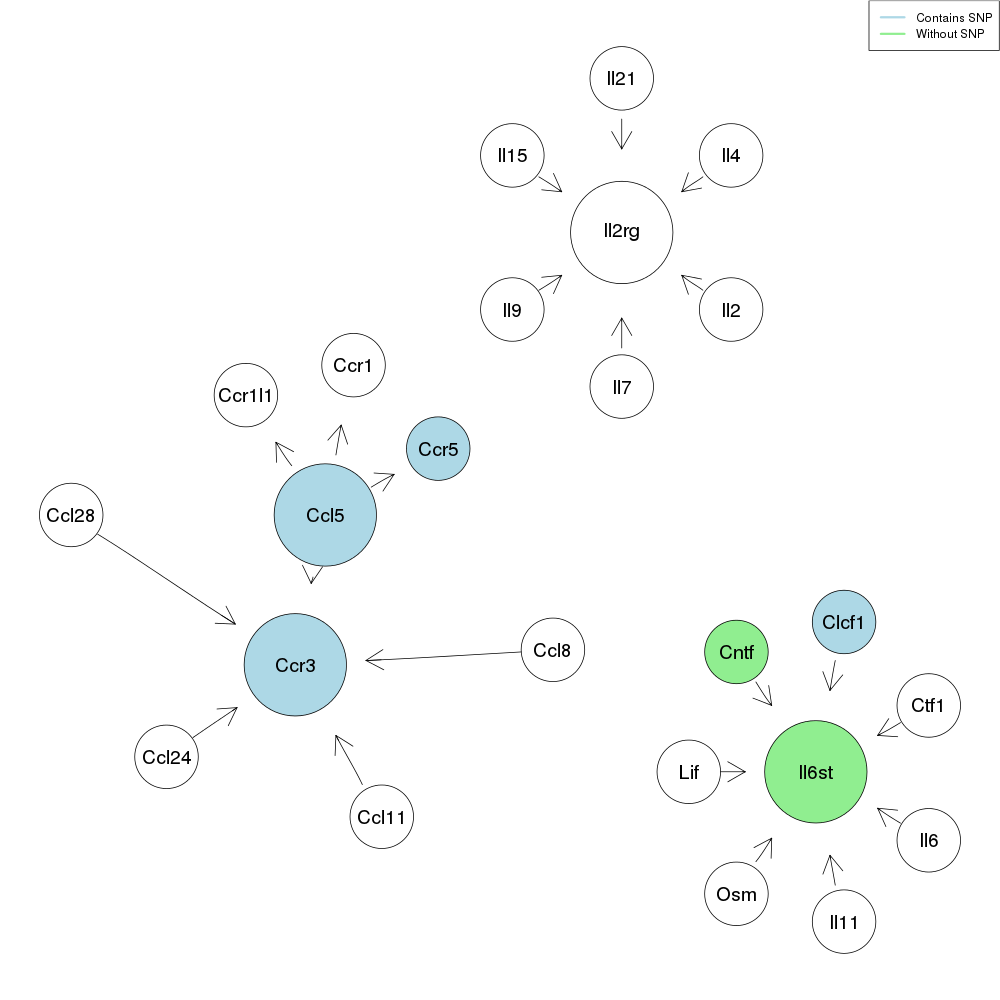

Supplement: Figure S5 — Cytokine-cytokine receptor interaction. (TIF) [file pone.0048472.s005.tif]

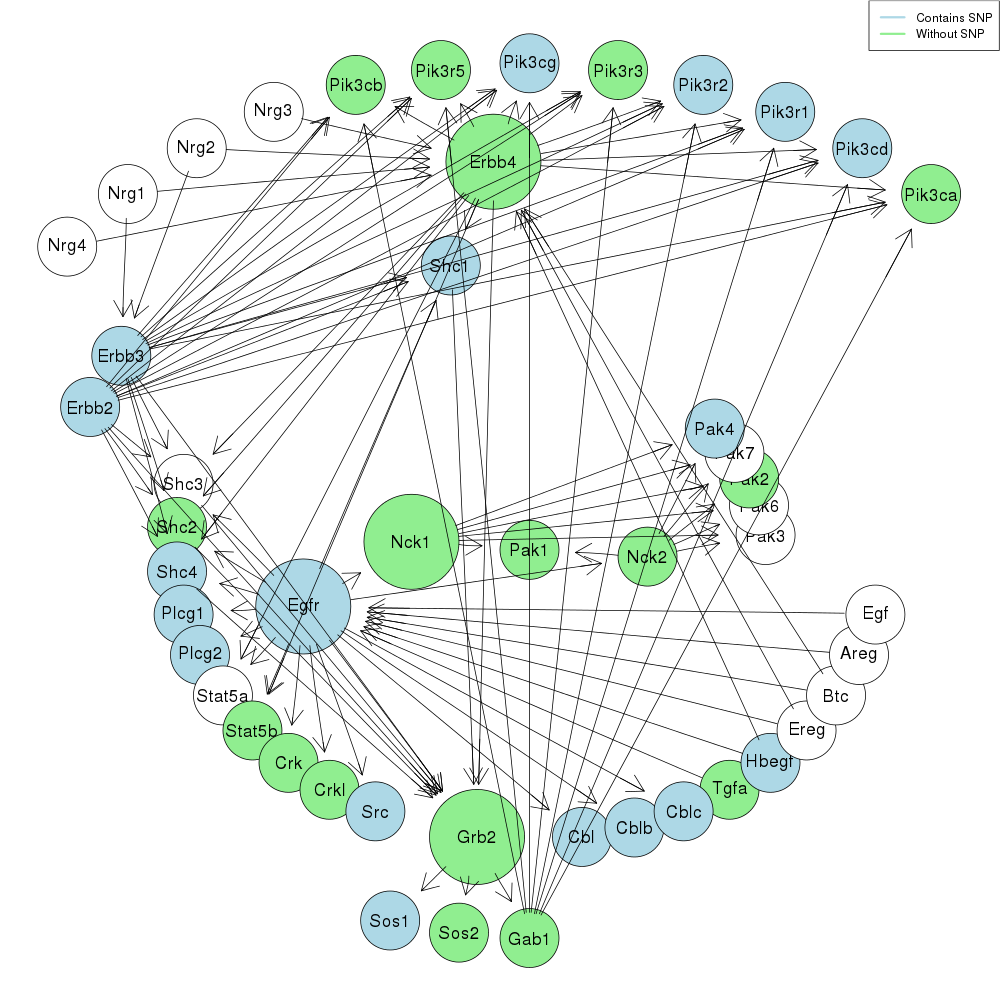

Supplement: Figure S6 — ErbB signalling pathway (TIF) [file pone.0048472.s006.tif]

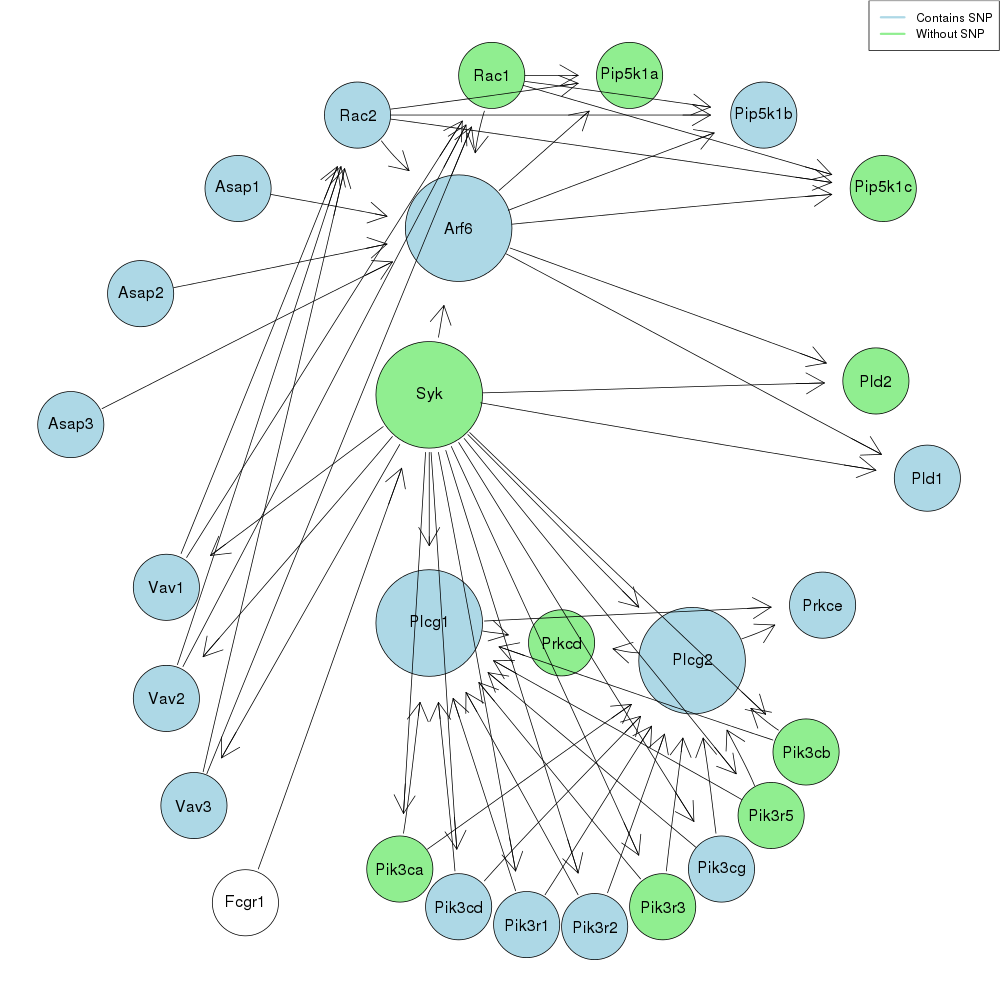

Supplement: Figure S7 — Fc gamma receptor-mediated phagocytosis. (TIF) [file pone.0048472.s007.tif]

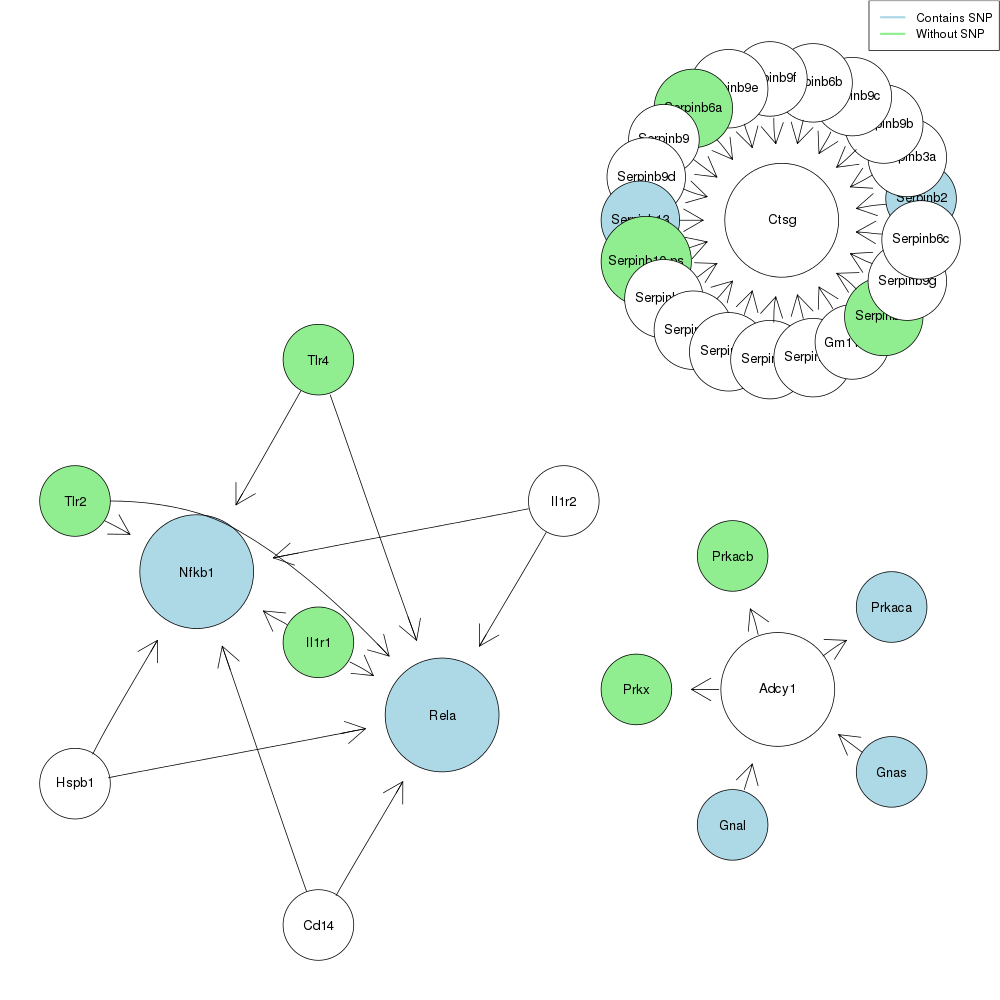

Supplement: Figure S8 — Intestinal immune network for IgA production. (TIF) [file pone.0048472.s008.tif]

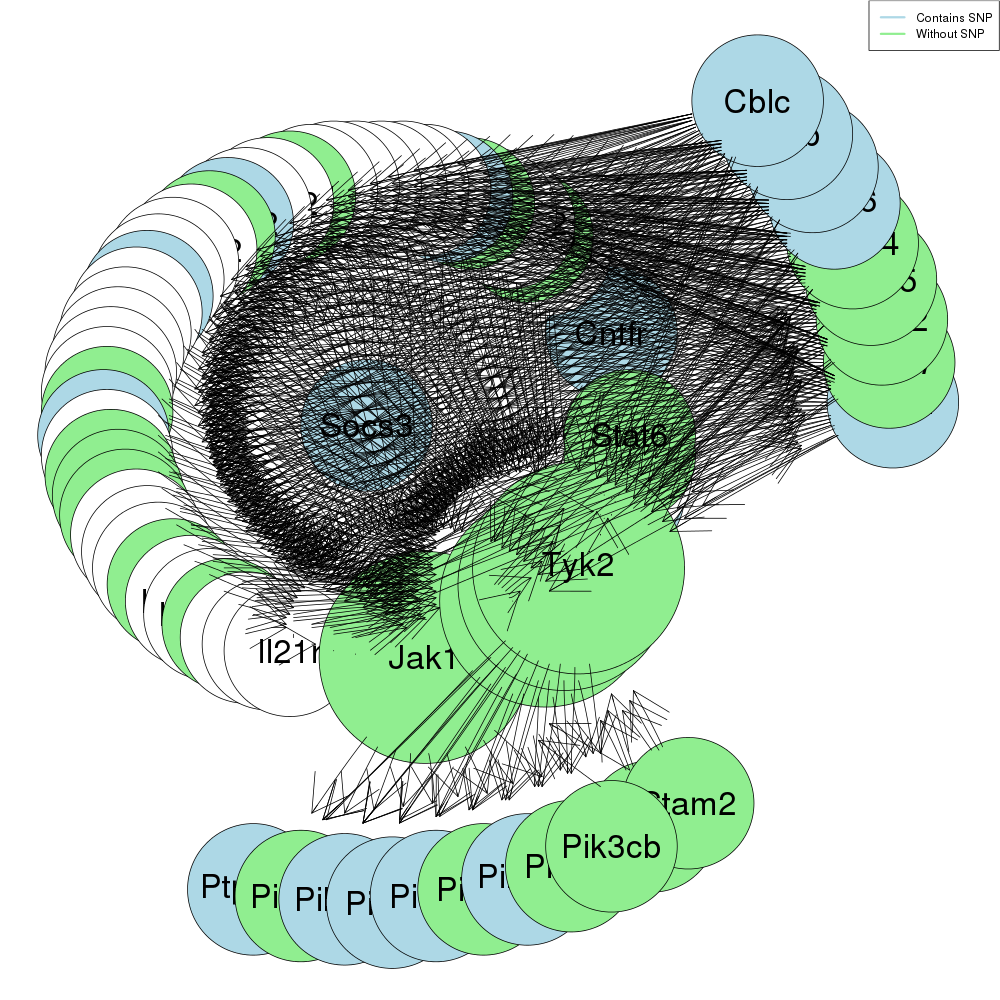

Supplement: Figure S9 — Jak-STAT signalling pathway. (TIF) [file pone.0048472.s009.tif]

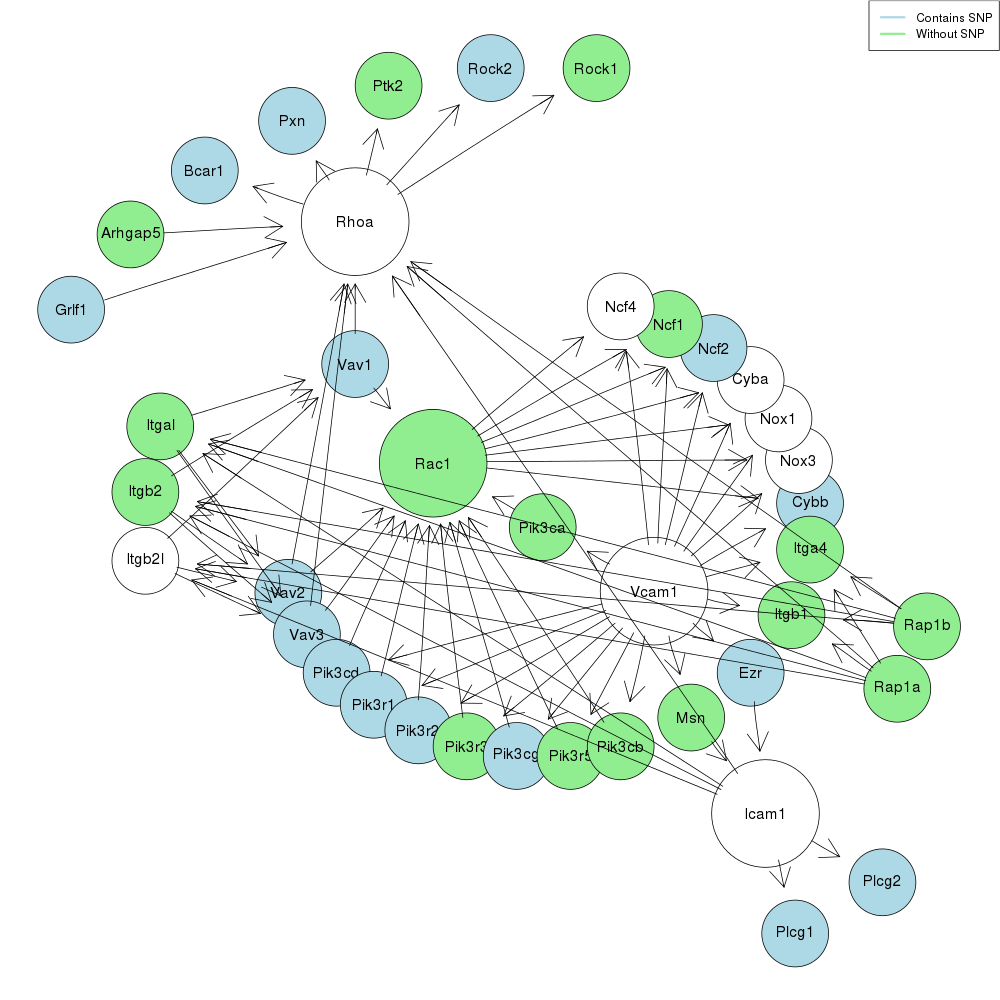

Supplement: Figure S10 — Leukocyte transendothelial migration. (TIF) [file pone.0048472.s010.tif]

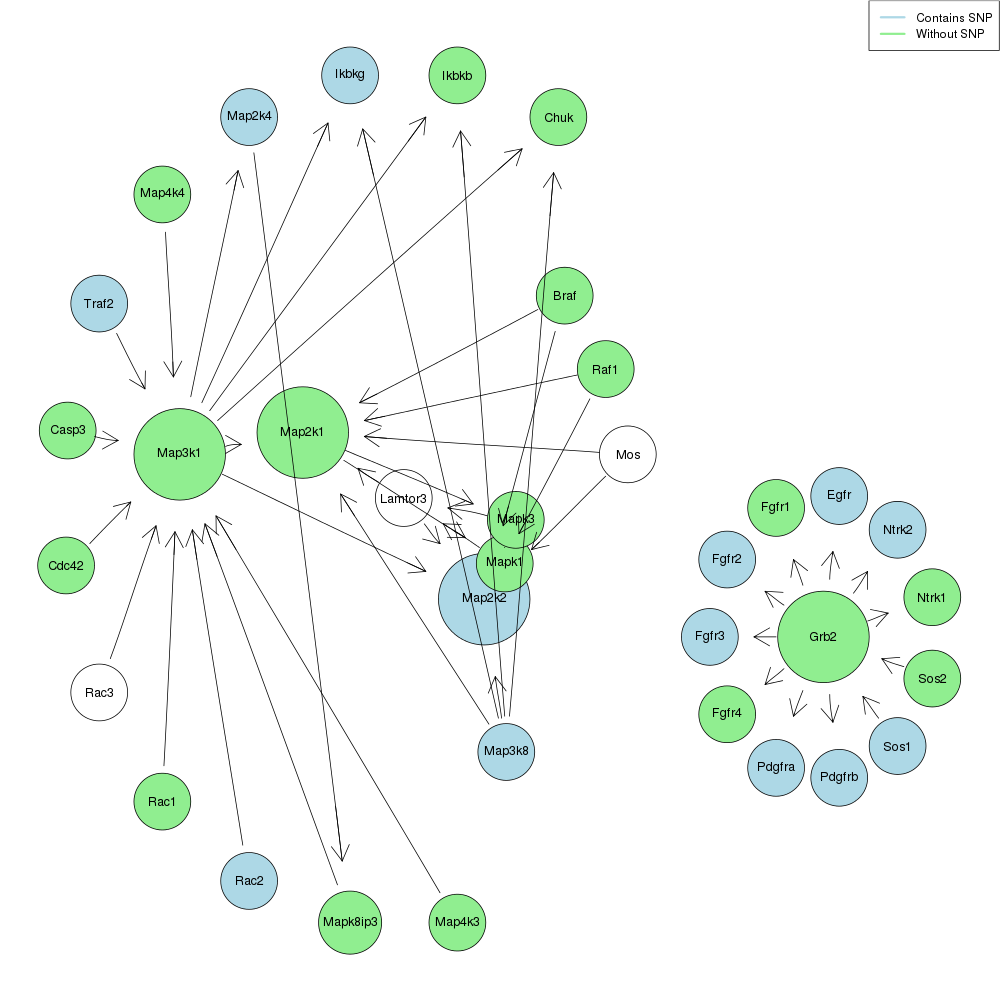

Supplement: Figure S11 — MAPK signalling pathway. (TIF) [file pone.0048472.s011.tif]

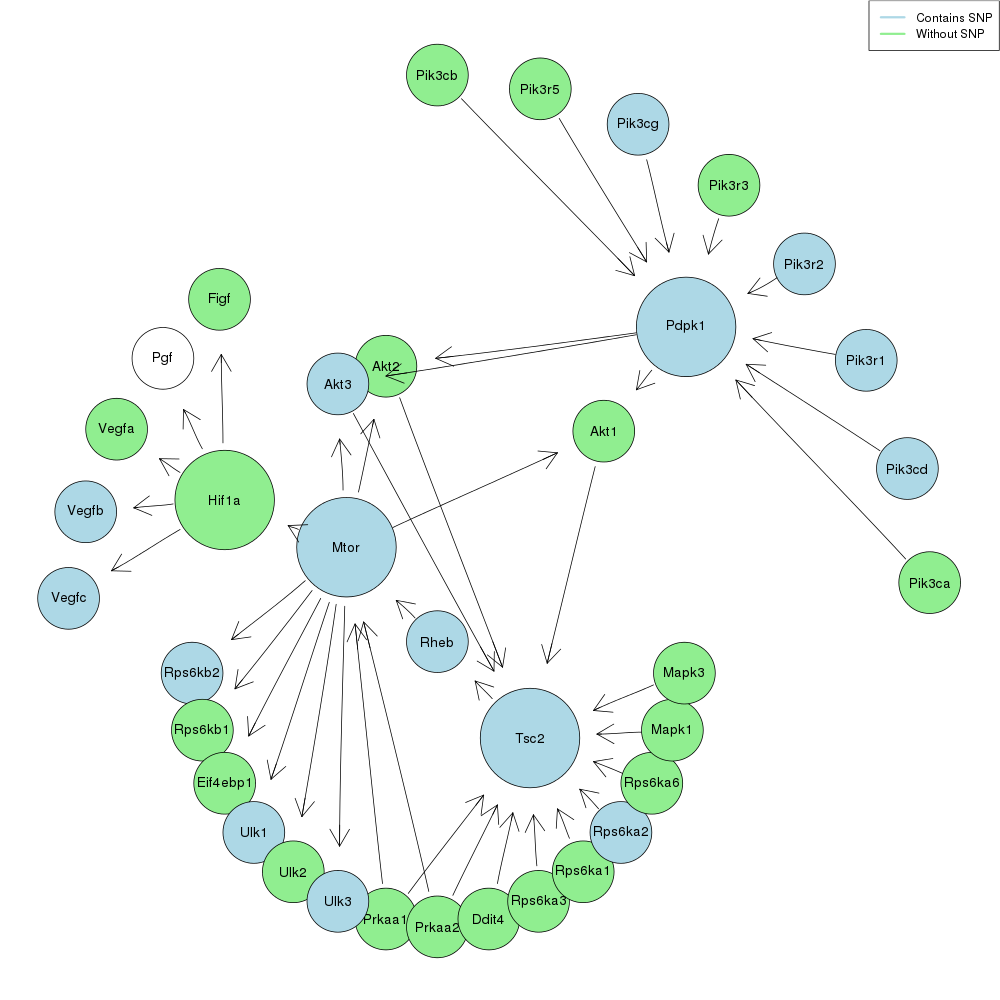

Supplement: Figure S12 — mTOR signalling pathway. (TIF) [file pone.0048472.s012.tif]

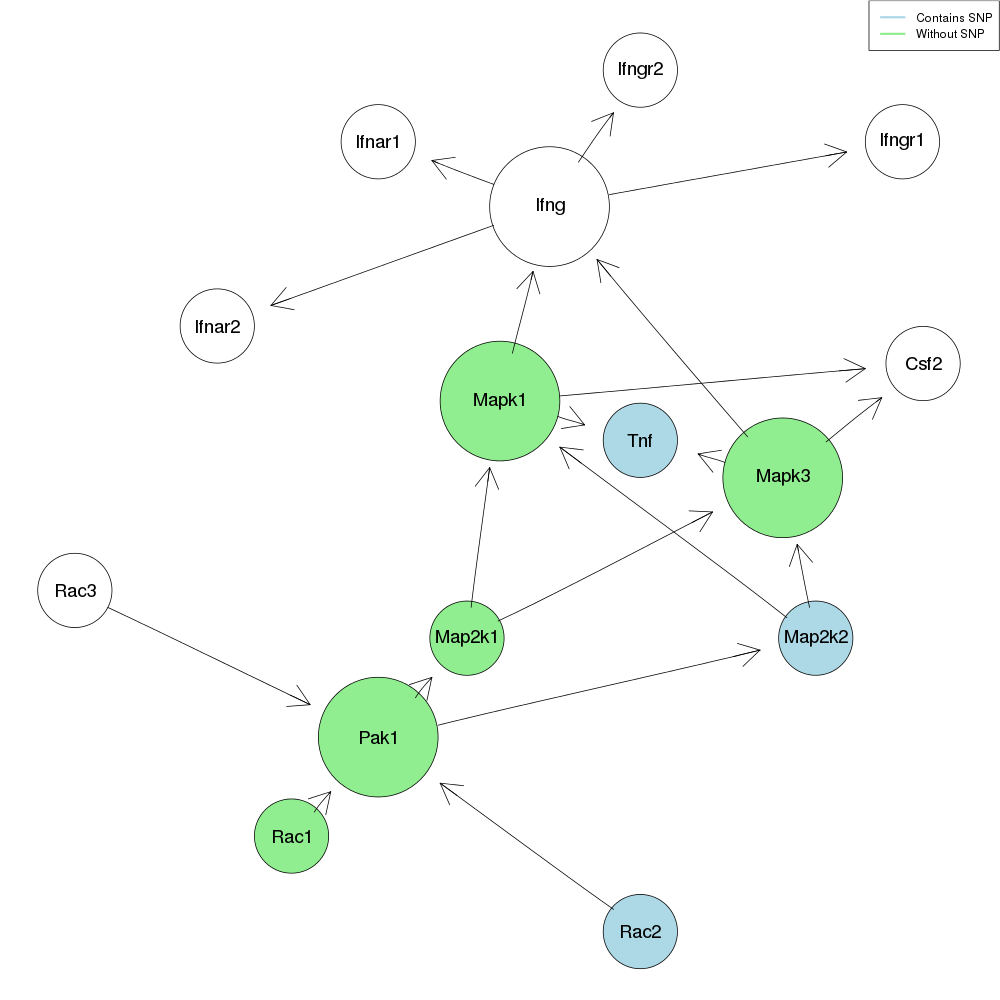

Supplement: Figure S13 — Natural killer cell-mediated cytotoxicity. (TIF) [file pone.0048472.s013.tif]

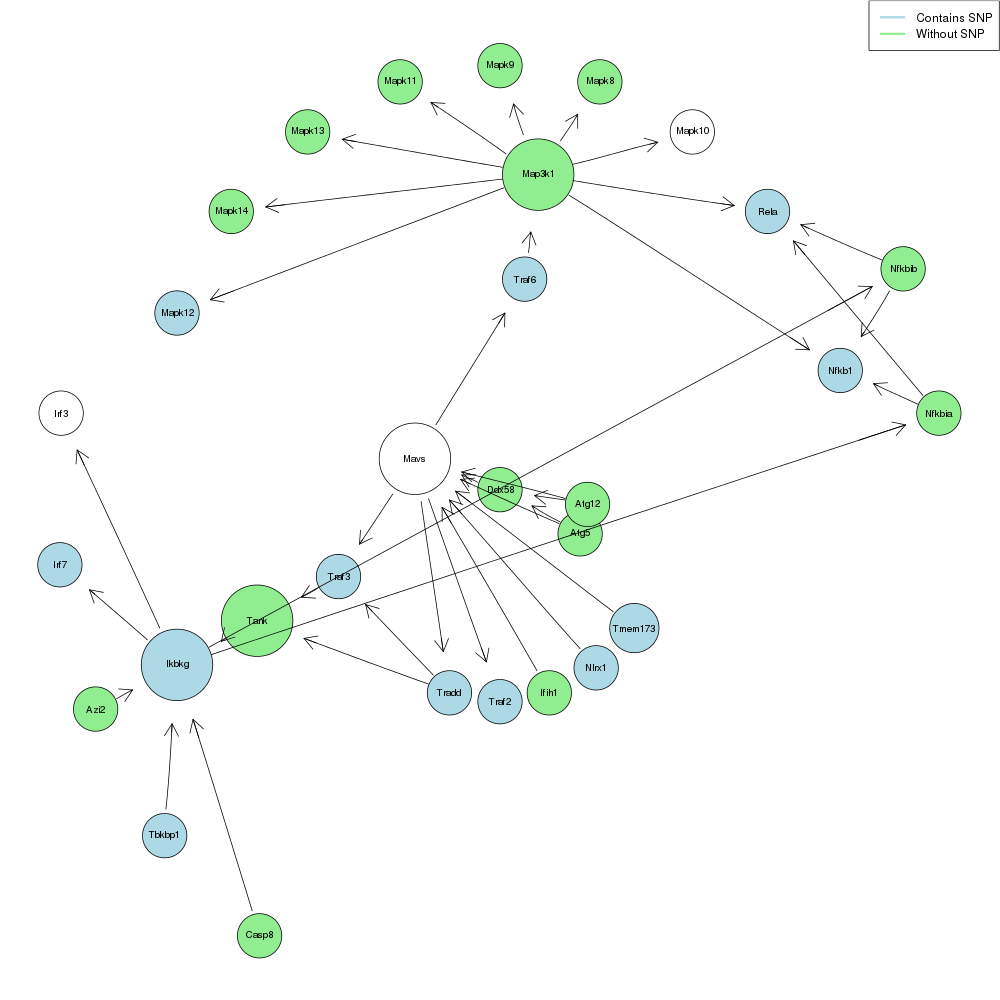

Supplement: Figure S14 — RIG-I-like receptor signalling pathway. (TIF) [file pone.0048472.s014.tif]

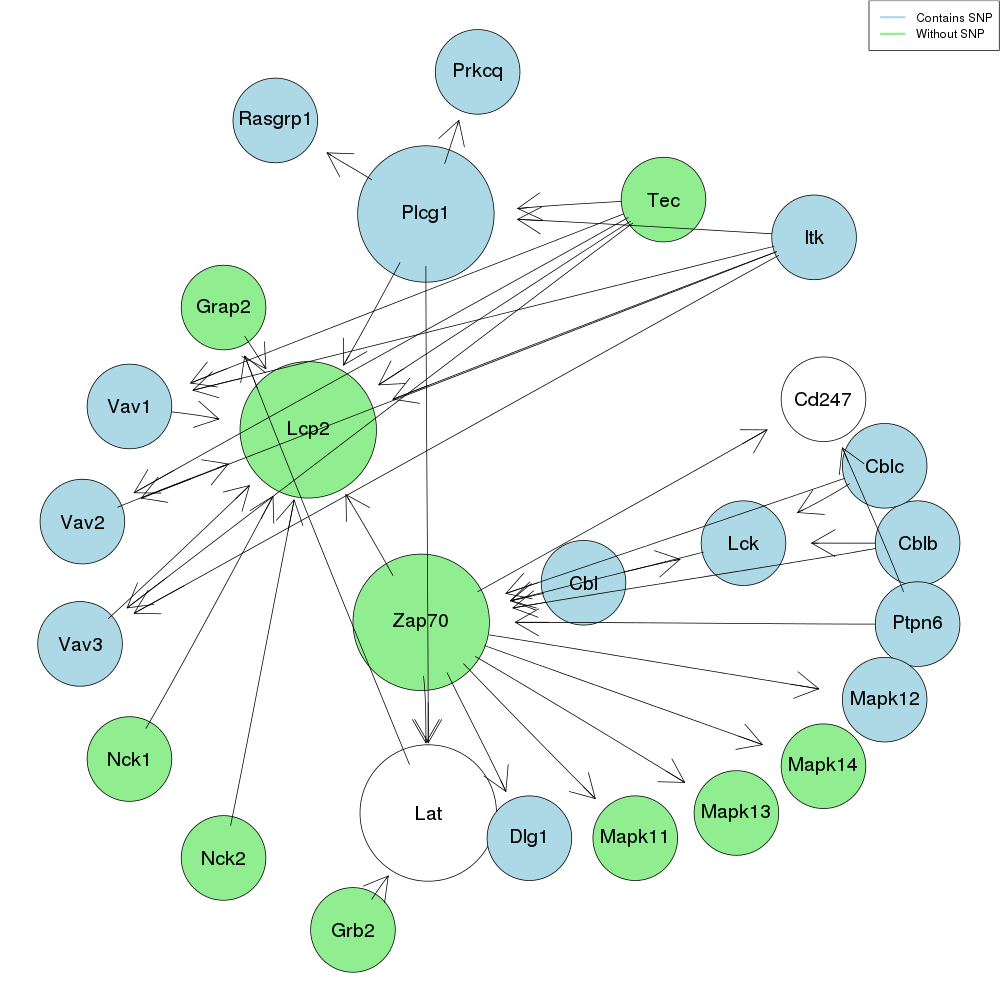

Supplement: Figure S15 — T cell receptor signalling pathway. (TIF) [file pone.0048472.s015.tif]

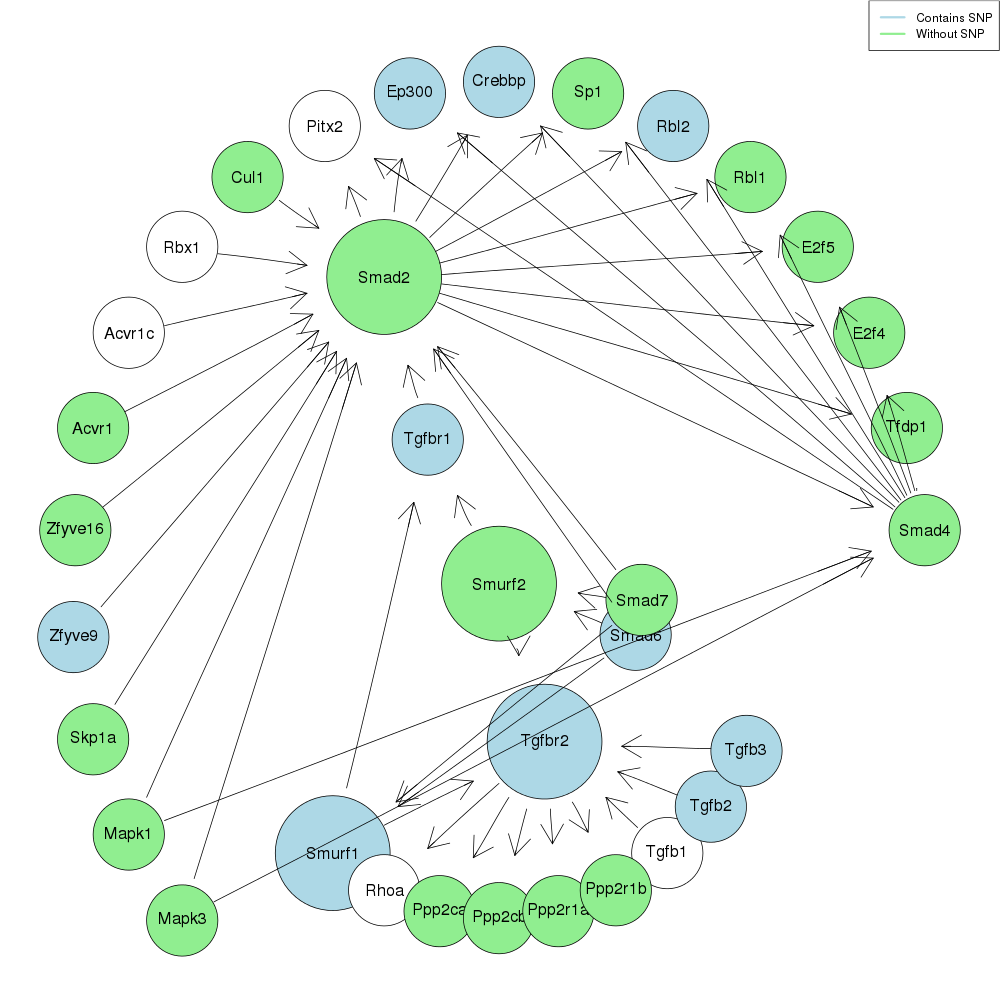

Supplement: Figure S16 — TGF beta signalling pathway. (TIF) [file pone.0048472.s016.tif]

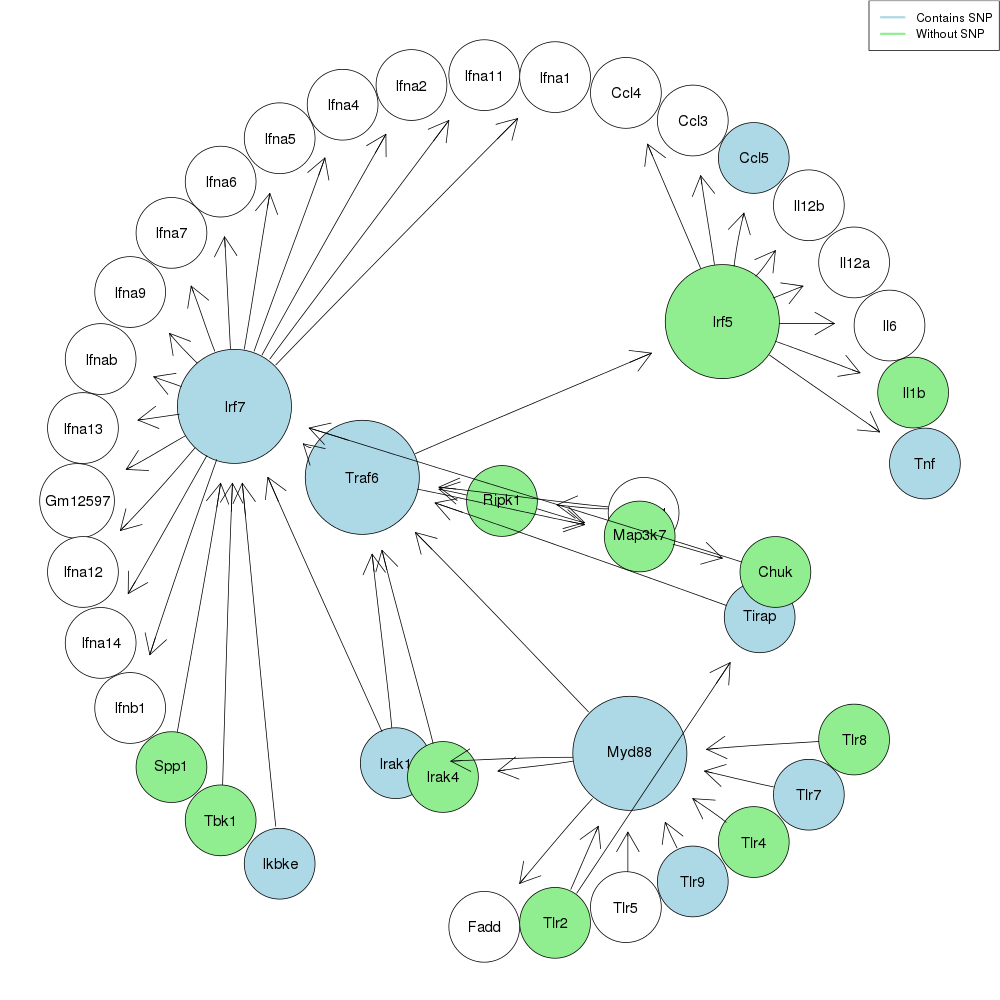

Supplement: Figure S17 — Toll-like receptor signalling pathway. (TIF) [file pone.0048472.s017.tif]

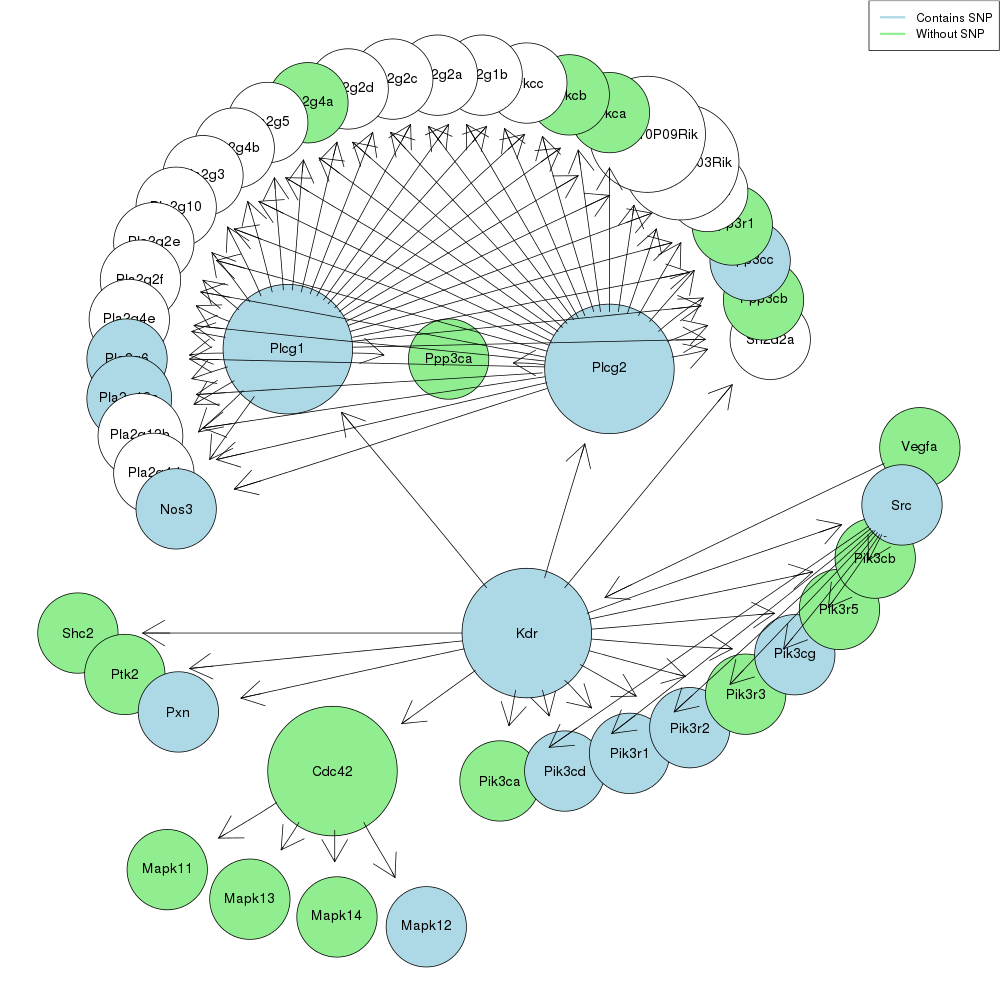

Supplement: Figure S18 — VEGF signalling pathway. (TIF) [file pone.0048472.s018.tif]
